# The tip of the iceberg: A call to embrace anti-localizationism in human neuroscience research

**DOI:** 10.1162/imag_a_00138

**Published:** 2024-04-18

**Authors:** Stephanie Noble, Joshua Curtiss, Luiz Pessoa, Dustin Scheinost

**Affiliations:** Department of Psychology, Northeastern University, Boston, MA, United States; Department of Bioengineering, Northeastern University, Boston, MA, United States; Center for Cognitive and Brain Health, Northeastern University, Boston, MA, United States; Department of Radiology and Biomedical Imaging, Yale School of Medicine, New Haven, CT, United States; Department of Applied Psychology, Northeastern University, Boston, MA, United States; Department of Psychiatry, Massachusetts General Hospital, Boston, MA, United States; Department of Psychology, University of Maryland, College Park, MD, United States; Maryland Neuroimaging Center, University of Maryland, College Park, MD, United States; Department of Biomedical Engineering, Yale University, New Haven, CT, United States; Interdepartmental Neuroscience Program, Yale University, New Haven, CT, United States; Department of Statistics and Data Science, Yale University, New Haven, CT, United States; Child Study Center, Yale School of Medicine, New Haven, CT, United States

**Keywords:** reductionism, cluster, complex systems, neuroimaging, philosophy of science

## Abstract

Human neuroscience research remains largely preoccupied with mapping distinct brain areas to complex psychological processes and features of mental health disorders. While this reductionist and localizationist perspective has resulted in several substantive contributions to the field, it has long been viewed as only a piece of the puzzle. Emerging evidence now empirically demonstrates how a historical reliance on localizationist techniques may underlie recent challenges to reproducibility and translation in human neuroscience. To advance discovery, we must collectively better incorporate complex systems and machine-learning approaches that better capture the multidimensional, dynamic, and interacting nature of the brain. Moreover, we must begin to contend with how to best integrate complementary modalities beyond the brain to better understand complex mental processes.

## 1 Introduction

This past year sparked widespread conversation and debate about how and whether we should map function to individual brain areas in humans. We witnessed a vigorous debate between experts in brain, mind, and philosophy at a symposium aptly named “The Brain is Complex: Have we Been Studying it all Wrong?” at the leading conference on cognitive neuroscience (i.e., the Cognitive Neuroscience Society meeting). A Special Focus issue “The Entangled Brain” (Postle, 2023) (named after the eponymous book;Pessoa, 2023) released a dizzying array of additional perspectives weighing in on this debate concerning the utility of complexity-oriented approaches (i.e., nonlinear, multivariate, and dynamic models). Leading neuroimaging statisticians designed an educational course “Beyond Blobology” at the major functional neuroimaging conference, the Organization for Human Brain Mapping meeting. The debate even pushed the boundaries of conventional venues: a pro-complexity opinion piece (Westlin et al., 2023) stirred spirited debate across the community on social media (the associated post by Feldman-Barrett was viewed more than 700,000 times, and a counterpoint by Kanwisher was viewed more than 500,000 times, with more than 150 unique comments added by the community).

The polarization of responses has been stunning. Many have shared strong opinions regarding whether to center complexity-oriented approaches to characterizing the brain or whether this shortchanges approaches geared towards uncovering specialized processes—and, in the midst of all this, meta-complaints about “strawman arguments” or that this is “not a new issue.” Indeed, this is a centuries-old debate (Mundale, 2002), but one, as we will argue below, that is deserving of its renewed attention given how historical limitations in computational and experimental methodology have shaped our current conceptual and empirical understanding of the human brain. These effects have important resounding implications for current research. Here, we provide a brief overview of how aspects of this debate have evolved in the context of historical human neuroscience research, how historical goals and practical limitations have shaped this research, where this leaves us today, and suggestions for a path forward^1^. Throughout, we draw from philosophical, historical, computational, and clinical perspectives.

## 2 Early Excitement about Localizing Biomarkers and Mechanisms in the 20 ^th^ Century

Human brain researchers frequently report findings that map cognitive function to specific underlying brain areas. This approach has a long history. In the 19^th^and 20^th^century—before quantitative measurement of the living human brain became a reality—we gained empirical insight into brain function by observing behavioral changes following focal impairment. From linking the hippocampus to memory to linking Broca’s and Wernicke’s areas to language, early evidence hinted at the tantalizing notion that many brain functions may find a nearly one-to-one mapping to brain areas (Savoy, 2001). Of course, cognitive scientists and philosophers have had just as long of a history of questioning the extent to which this perspective reflected reality and what else may be missing. However, by the advent of contemporary neuroimaging technologies in the late 20^th^century,*localizationism*was by far the predominant approach.

With the emergence of quantitative imaging and sensor technologies for measuring brain signals (i.e., neuroimaging) in the 20^th^century, excitement grew about the potential for tangible insight into problems that had long eluded us (Savoy, 2001). Advances in imaging and sensor technology presented an opportunity to probe brain function with unprecedented spatial and temporal precision. An explosion of research programs emerged globally to identify neural bases and biomarkers of behavior and disease, often in the form of spatially specific brain areas, tracts, and local circuits. Despite ongoing debates, the bulk of empirical human neuroscience research by and large reflected an implicit*ontological reductionist*perspective in the field: the idea that mental processes can be reduced to and are identical to neurobiological processes.^2^

Researchers studying brain disease were particularly motivated to follow the successes in other areas of medicine. Biological reductionism became considerably associated with the idea that complex behavioral phenomena are the consequence of relatively simple underlying biological mechanisms. The 20^th^century resulted in significant successes in identifying simple, single causes of physical pathologies, such as linking cardiac angina to arterial obstructions and cancers to resectable tumors. This reductionist medical model provided a reasonable explanatory account of specific diseases for which simple, localized causes—disease mechanisms—could be ascertained. Consequently, the*medical model*also came to dominate psychiatry as an aspirational model for understanding and treating disease (Nesse & Stein, 2012). Accordingly, the nation's top funder of health-related research, the National Institutes of Health (NIH), prioritized funding for identifying biological causes of mental illness and neurological disease, which shaped the perspective of many in the field. This effort was buoyed by the emergence of the ambitious Brain Research through Advancing Innovative Neurotechnologies (BRAIN) Initiative in 2013, a nationwide public-private partnership that revitalized funding for all neuroscience research. Importantly, despite ostensible statements about the importance of understanding the interactivity and dynamic nature of the brain, language used by funders and researchers alike often harkened back to reductionist concepts like finding local circuits, collections of genes, etc.—perspectives that were, in turn, echoed by countless projects and manuscripts that sought to link findings to circumscribed brain areas and local circuitry. For example, the Research Domain Criteria (RDoC) Matrix, which illustrates guiding principles for mental health research, predominately enumerates specific biological mechanisms such as localized regions and circuits as the most relevant link to mental health disorders (Cuthbert & Insel, 2013).

Thus, it is no surprise that methods designed to identify focal brain areas and circuitry underlying behavior and disease quickly became the bread and butter of research in human neuroscience (e.g., mass univariate inference). Their interpretability made them a great match for the problems researchers were interested in solving, as they allowed for concrete brain models to be hypothesized and tested. Perhaps as important, such methods were computationally efficient, a necessity for the relatively limited computing capabilities of much of the 20^th^century. This localizationist perspective has provided essential insights into the brain, most notably illuminating key players in several psychological and neurological processes (e.g., motor control, language). It has even shown promise for some applications, such as neurofeedback and surgical planning (Benjamin et al., 2017). Yet while localizing computational methods are now among the most common approaches used in the field, a longstanding and growing undercurrent of dissatisfaction with such approaches has been urging more researchers to consider whether we can do better in how we conceptualize human brain mapping of complex psychological processes (Neuroskeptic, 2012;Tononi et al., 1994).

## 3 A Crisis and a Turning Point

While human neuroscience has witnessed several decades of exciting advances, they have not led to the explosion of insight into the brain bases of complex human behavior and disease that scientists expected. After a flourishing early era of well-funded exploration, the field has faced ongoing challenges about the integrity and translational potential of our body of research. Many of these concerns have fallen under the umbrella of an oft-bemoaned “reproducibility crisis.” A recent flurry of reports has demonstrated that many typical research findings reflect only modest associations with psychological processes, low power, and poor reliability (e.g.,Button et al., 2013;Marek et al., 2022;Noble et al., 2022). Considering these issues, it is perhaps unsurprising that clinical translation has been slow or rather modest (Barry, 2022;Schleim & Roiser, 2009;Seyhan, 2019) (note similar concerns rising in preclinical neuroscience; cf.Spanagel, 2022).

Issues with reliability and validity stem from a number of suboptimal research practices that are understandable in the context of an early, exploratory phase of modern human neuroscience research (e.g., emerging standards for study design). However, researchers have pointed out that these issues can be attenuated when we move beyond mass univariate approaches designed for localization and instead model more widespread and complex signals (e.g.,Kragel et al., 2021;Marek et al., 2022;Noble et al., 2017,^2022^). Accordingly, the idea that we may not be able to neatly define discrete brain mechanisms underlying behavior has begun to spread more seriously throughout the broader human neuroscience community. Exactly what the alternative looks like remains an open question, but there is certainly abundant evidence against a simple one-to-one mapping for many psychological phenomena. A contemporary understanding of this evidence as well as the tenets underlying localizationism and*anti-localizationism*has been appositely distilled byMcCaffrey (2023), which we briefly review here.

**Fig. 1.**
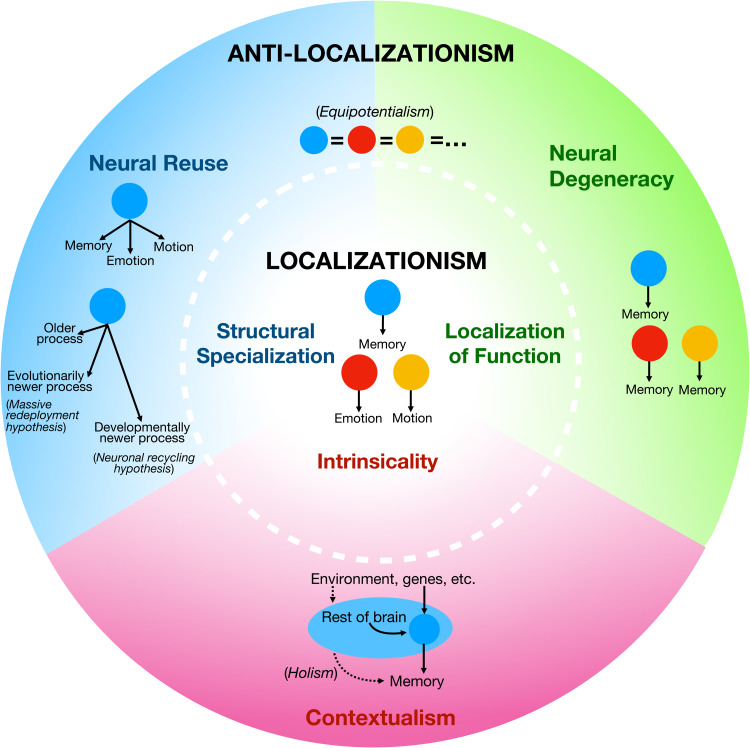
A contemporary conceptualization of localizationist and anti-localizationist tenets, following the framework introduced byMcCaffrey (2023). Colored circles indicate different brain areas or circuits. The boundary between localizationism and anti-localizationism illustrates the mutually exclusive distinction between these concepts in the metaphysical context (i.e., in terms of reductionism and anti-reductionism), whereas the gradient from the center to the boundary of the circle illustrates the gradual transition between the extremes of these concepts in the empirical context.

The three identified pillars of contemporary localizationist and anti-localizationist viewpoints include, respectively:*Specialization*versus*Neural Reuse*,*Localization of Function*versus*Neural Degeneracy*, and*Intrinsicality*versus*Contextualism*(Fig. 1). In brief, evidence from multiple studies and meta-analyses presently suggests that brain areas are more likely to be involved in multiple cognitive domains (i.e., Neural Reuse) than a single one (i.e., Specialization). Neural Degeneracy arguments point towards the existence of multiple different systems that support the same function; for example, the ability to rebuild vision or language function after damage (e.g., lesion or surgery) to function-serving tissue, as well as inter- and intra-individual differences in brain mechanisms underlying the same cognitive function. Finally, arguments for Contextualization dissect the functions of various areas—and, indeed, neurons within those areas—to show that their behavior is inherently driven by environmental demands, the state of the brain network in which they are a part, and so on. As we contemplate Contextualization, we are led to also consider*holism*, the idea that the whole system of the brain (or much of it) may need to be considered simultaneously in order to understand some psychological processes. In fact, conventional applications of univariate localizing methods^3^have been shown to permit only the most robust and straightforward component of a whole-brain (or nearly whole-brain) signal—the tip of the iceberg—to survive (Gonzalez-Castillo et al., 2012;Noble et al., 2022). Given the decades of research using methods to isolate brain regions, which then form the targets for subsequent research, which in turn use localizing methods for inference, and so on in a cyclical process, we can begin to appreciate how reliance on these methods has reinforced the primacy of localizationism in guiding human brain research. Altogether, the above empirical challenges imply that the localizationist literature often scratches the surface of understanding and at times points researchers in the wrong direction.

This perspective has led some to reconsider the utility of mining human brain data for insights into behavior and disease. The lack of progress after so much hope, time, and funding—especially for addressing urgent disease and disability—can be discouraging. Yet, the challenges brought on by widespread adoption of the localizationist perspective remain little discussed in the field. At this juncture, it may be profitable to reflect on the successes and shortcomings of our previous brain mapping strategies and explore a new way forward that adequately respects the complexity of the problem at hand.

## 4 Anti-Reductionism: a Philosophical Framework for Anti-Localizationism

It is critical to understand the philosophical underpinnings underlying this debate. Anti-localizationism relies on the philosophical scaffolding of*anti-reductionism*, which, at its heart in this context, is the idea that complex psychological processes may involve phenomena that cannot be reduced to discrete biological processes (i.e., there is no one-to-one mapping). A common analogy is the distinction between software and hardware in computer science. Notably, different hardware architectures can execute the same software program, underscoring the idea that the same abstract computational function can be implemented by diverse physical substrates. Philosophy and metaphysics have long indicated problems underlying biological reductionist approaches, including issues with*one-to-many*and*many-to-one*mappings. Although an extensive explication of these topics is beyond the purview of the current paper, both issues present significant obstacles to biological reductionism and have been discussed extensively in philosophy of mind literature (Brigandt & Love, 2023;Fodor, 1980). The one-to-many critique of reductionism underscores that a single lower-level feature can give rise to different higher-level phenomena depending on context (i.e., in genetics, the same allele may engender two different phenotypes if in two separate individuals with disparate genotype environments). Conversely, the many-to-one critique of reductionism stipulates that a given higher-level phenomena can be realized by different lower-level configurations and features, giving rise to*multiple realizability*(i.e., a single mental property or cognitive process, such as language comprehension, can be realized by several different types of lower-level, biological brain substrates). Multiple realizability presents a major obstacle to reductionist accounts of the mind and brain.^4^

Often, when localizationism and anti-localizationism are used in common language by neuroscientific researchers, there is an inadvertent conflation of the metaphysical and philosophical underpinnings of these terms with their descriptive, everyday meanings. That is, a metaphysical characterization concerned with the “ground truth” differs from an empirical or descriptive characterization, where we are more concerned with how things usually happen (cf.Fig. 1). While localizationism and anti-localizationism have a metaphysical foundation in terms of reductionism and anti-reductionism, the former terms are also commonly used to refer to the statistical frequency of association between brain and behavior measured empirically. Even if anti-reductionism obtains for a given psychological process, it may still be the case that certain brain regions are associated with that psychological process with some degree of statistical regularity. There may exist biological reasons that contribute to why certain brain regions are associated with particular psychological functions with statistical regularity (e.g., perhaps a certain structural configuration at the biological level may be well-suited to subserve particular computational functions that support particular forms of cognition). However, this does not necessarily entail that specific brain regions are a necessary condition for realizing certain cognitive, computational functions and therefore are reductionist in nature. Thus, it is important to emphasize that while localizationism may appear to hold in an empirical sense in some cases wherein specific brain regions are associated with specific cognitive functions with some statistical frequency, this in itself does not undermine the fact that those same cognitive processes may be best accounted for by an anti-reductionist framework at the “ground truth” metaphysical level.

Although it may seem unclear what implications a philosophical perspective has for empirical research, it is invaluable here because it forms a rigorous conceptual framework on which we can articulate our theories and test them empirically. In fact, conversations about reductionism and modularity have been guiding important discussions in the applied contexts of cognitive science and mental health for more than half a century (Barrett & Kurzban, 2006). Importantly, anti-reductionist positions have emerged repeatedly in these contexts recently in the face of reductionist optimism and funding (Borsboom et al., 2019;Buckner & Garson, 2019;Faucher & Goyer, 2015). Altogether, the substantive challenges to localizationism from the metaphysical perspective—reinforced by challenges from theoretical, empirical, and pragmatic perspectives—provide impetus to reconsider the status quo assumptions guiding experimental neuroscience research.

## 5 A Way Forward

How, then, to better account for this complexity? Here, we take a moment to clarify where we have drawn our own lines lie in the sand. Overall, based on the evidence summarized above, we endorse a largely anti-localizationist and anti-reductionist view for much of neuroscience. We hypothesize that at least partial holism may be a more serious contender for explaining brain function than has historically been appreciated, yet we do not go so far as to espouse*equipotentialism*(i.e., that all brain units are interchangeable; we do not expect that any serious neuroscientist endorses this position). Moreover, anti-localizationism does not preclude that certain cognitive processes may empirically display organization with a certain degree of specialization with some degree of statistical regularity, as discussed above. We anticipate organization along a spectrum following the massive redeployment (Anderson, 2010) and neuronal recycling (Dehaene, 2005) hypotheses. These hypotheses state that evolution and development repeatedly co-opt older tissue for newer cognitive needs. In turn, older cognitive functions may be easier to localize to specific brain areas than newer functions because there is more existing circuitry available as time progresses to support newly emerging functions (McCaffrey, 2023). (Note that this does not mean that older brain areas*exclusively*handle older functions; on the contrary, these hypotheses specifically entail that older brain regions play a role in newer cognitive processes, e.g.,Janacsek et al., 2022.) Accordingly, we expect more specialized circuitry for lower-level, bottom-up, and automatic processes—for example, the need for greater specialization to support critical sensory processes (e.g., retinotopic organization in visual cortex), as well as to react automatically to urgent demands (e.g., a human frantically fleeing an approaching bee, or an amphibian catching a fly).

We further emphasize a pragmatic expectation that there may be some contexts in which studying areas in relative isolation can provide meaningful added or primary insight, depending on the processes of interest (Druckmann & Rust, 2023;Van Bouwel et al., 2011). However, even relatively well-defined lower-level psychological processes can exhibit surprisingly complex features, so it may be prudent to take care when studying processes in isolation even in these contexts. We anticipate that recent technological and conceptual advances will enable us to more explicitly articulate and test the extent to which brain functional organization may empirically exhibit properties that lie between the extremes of localizationism and anti-localizationism.

Taking together the historically skewed emphasis on localizationism alongside the theoretical and pragmatic arguments for anti-localizationism, we advocate for a greater emphasis on anti-localizationism across human neuroscience research. We see two guiding principles: 1) the broader use of computational frameworks designed for better capturing and modeling brain complexity, and 2) the adoption of frameworks that integrate multiple levels of explanation (Table 1).

**Table 1.** Guidelines for adopting anti-localizationist practices in human neuroscience research.

Approach	Motivation	Examples	Key references
**Modeling Complexity I:** Complex Systems	*Theoretically motivated* investigation of multidimensional, nonlinear, and interacting neuroscience phenomena	Relevant topics include network science and control theory, high dimensional geometry, dynamical systems, etc.	Boccara, N. Modeling Complex Systems. 1st 2004. edn, (Springer New York: Imprint: Springer, 2004). https://doi.org/10.1007/978-1-4419-6562-2 Bassett, D. S. & Sporns, O. Network neuroscience. Nat Neurosci 20, 353-364 (2017). https://doi.org:10.1038/nn.4502 Lurie, D. J. et al. Questions and controversies in the study of time-varying functional connectivity in resting fMRI. Netw Neurosci 4, 30-69 (2020). https://doi.org:10.1162/netn_a_00116 Shine, J. M., et al. Human cognition involves the dynamic integration of neural activity and neuromodulatory systems. Nature neuroscience 22.2 (2019): 289-296. https://doi.org/10.1038/s41593-018-0312-0 Gao, S., Mishne, G. & Scheinost, D. Nonlinear manifold learning in functional magnetic resonance imaging uncovers a low-dimensional space of brain dynamics. Hum Brain Mapp 42, 4510-4524 (2021). https://doi.org:10.1002/hbm.25561
**Modeling Complexity II:** Machine Learning	*Data-driven* identification of brain-behavior relationships with varying degrees of a priori constraints on complexity	Common approaches include mass univariate filtering, regularized regression, random forest, support vector machines / regression, deep learning, etc.	Davatzikos, C. Machine learning in neuroimaging: Progress and challenges. Neuroimage, 197, 652. (2019). https://doi.org/10.1016/j.neuroimage.2018.10.003 Varoquaux, G., et al. Assessing and tuning brain decoders: cross-validation, caveats, and guidelines. NeuroImage 145 (2017): 166-179. https://doi.org/10.1016/j.neuroimage.2016.10.038 Pervaiz, U., et al. Optimising network modelling methods for fMRI. Neuroimage 211 (2020): 116604. https://doi.org/10.1016/j.neuroimage.2020.116604 Scheinost, D. et al. Ten simple rules for predictive modeling of individual differences in neuroimaging. Neuroimage 193, 35-45 (2019). https://doi.org:10.1016/j.neuroimage.2019.02.057 Lundervold, A. S. & Lundervold, A. An overview of deep learning in medical imaging focusing on MRI. Z Med Phys 29, 102-127 (2019). https://doi.org:10.1016/j.zemedi.2018.11.002
**Integrating Levels I:** Cross-level Modeling	Complex psychological processes cannot be reduced to simple biological processes; biological processes can be intrinsically related to other levels of explanation	Analyses with a diverse set of variables, including gene, brain, psychological, sociocultural, and environmental sources, etc.	Greene, A. S. et al. Brain-phenotype models fail for individuals who defy sample stereotypes. Nature 609, 109-118 (2022). https://doi.org:10.1038/s41586-022-05118-w Li, J., et al. Cross-ethnicity/race generalization failure of behavioral prediction from resting-state functional connectivity. Science Advances 8.11 (2022): eabj1812. https://doi.org/10.1126/sciadv.abj1812 Dadi, K. et al. Population modeling with machine learning can enhance measures of mental health. Gigascience 10 (2021). https://doi.org:10.1093/gigascience/giab071 Alfaro-Almagro, F., et al. Confound modelling in UK Biobank brain imaging. NeuroImage 224 (2021): 117002. https://doi.org/10.1016/j.neuroimage.2020.117002
**Integrating Levels II:** Interdisciplinary collaboration	Facilitate cross-level modeling; multiple fields are facing similar problems regarding reductionism and complexity and can benefit from similar solutions	Collaboration across fields, including neuroscience subdisciplines, genetics, psychology, psychiatry, etc.	Machado, T. A., Kauvar, I. V. & Deisseroth, K. Multiregion neuronal activity: the forest and the trees. Nature Reviews Neuroscience, 1-22 (2022). https://doi.org/10.1038/s41583-022-00634-0 Tremblay, S., et al. Non-necessary neural activity in the primate cortex. bioRxiv, 2022.2009.2012.506984 (2022). https://doi.org:10.1101/2022.09.12.506984 Pu, S., Dang, W., Qi, X.-L. & Constantinidis, C. Prefrontal neuronal dynamics in the absence of task execution. bioRxiv, 2022.2009.2016.508324 (2022). https://doi.org:10.1101/2022.09.16.508324 van der Meer, D. et al. Understanding the genetic determinants of the brain with MOSTest. Nature Communications 11, 1-9 (2020). https://doi.org/10.1038/s41467-020-17368-1 Glazer, N. L. Moving beyond genome-wide association studies. Circ Cardiovasc Genet 4, 91-93 (2011). https://doi.org:10.1161/CIRCGENETICS.110.958785 Hofmann, S. G., Curtiss, J. & McNally, R. J. A Complex Network Perspective on Clinical Science. Perspect Psychol Sci 11, 597-605 (2016). https://doi.org:10.1177/1745691616639283

Motivation and examples are provided for approaches that modeling complexity and integrating levels of explanation in research practice.

Frameworks for capturing complexity may involve varying degrees of theory. The umbrella field for the more theory-driven study of complex phenomena is called*complex systems*(Boccara, 2004;Scheffer, 2020). Complex systems can mean many things. Just as is the case for reductionism, an underlying principle is that a complex system exhibits “emergent” collective properties that cannot be deduced from its parts^5^. In short, the many branches of this field come together to capture multidimensional, embedded, nonlinear, and interacting phenomena as they unfold over time. Popular neuroimaging applications draw from many of these branches, including network science (e.g., functional and structural connectivity) and control theory (Bassett & Sporns, 2017), high dimensional geometry (Gao et al., 2021), dynamical systems (Lurie et al., 2020), and more. Overall, the resolutely multivariate nature of complex systems reflects the reality of neuroscience data; in turn, multivariate approaches alone improve power and reproducibility compared with univariate approaches (Marek et al., 2022;Noble et al., 2022).

These approaches can be used to characterize the brain in different states and contexts, including in response to stimuli (e.g., from tasks or direct perturbation). Direct perturbation offers an unparalleled opportunity to gain a deeper understanding of the system's constraints and behavior—a perspective that may remain elusive through other means. Simultaneously, it is important to acknowledge the limitations of ascribing results of perturbation to single areas (Barack et al., 2022). Manipulating elements of a system (e.g., through electrical stimulation) may not reveal the full extent of a complex network’s involvement in a process but instead may reveal relatively critical components. Even perturbing a single focal area may affect the whole system in a complex way that makes results difficult to interpret—that is, observed results may be difficult to uniquely reduce to the single area. Bearing this perspective in mind, the exploration of neural perturbations alongside other methods has strong potential to enrich our understanding of the brain as a complex system.

On the less theory-driven side, many*machine-learning*approaches aim to derive complementary representations of phenomena. While these can be used in conjunction with complex systems characteristics, machine learning can also help avoid the challenging task of characterizing complex features*a priori*by instead learning difficult-to-define brain-behavior relationships. There has been a surge of machine-learning applications in neuroimaging that range from simple mass univariate approaches to less dissectible deep learning approaches that capture signal change over time and space; additional commonly used approaches include regularized regression, random forest, and support vector machines (Davatzikos, 2019;Scheinost et al., 2019).

A less examined problem is how*multiple levels of explanation*can be fruitfully combined. Researchers often take the perspective of treating physiological or environmental variables as confounds to be removed in order to “isolate” neural mechanisms. However, there is a growing focus on how brain function and disease is dependent upon the physiological and environmental contexts within which it is immersed (Bolt et al., 2023;Kendler, 2005;Sadaghiani & Alderson, 2023;Westlin et al., 2023). Furthermore, recent work illustrates how social and environmental factors can act as an intrinsic scaffolding necessary for the creation of brain-behavior models (Greene et al., 2022). Since most cognitive processes involve multiple levels, it may be safest to adopt a perspective of*compatible explanatory pluralism*(i.e., considering the existence of multiple levels of explanations to better reflect the complexity of multilevel systems, with the idea of ascertaining an integrative explanation of an overall complex system;Ludwig & Ruphy, 2021;Massimi, 2022) in the interim and we suggest considering the investigation of multiple levels simultaneously (Dale et al., 2009). A*multi-level*, or, indeed,*cross-level approach*is needed, which requires bridging domain-specific expertise with interdisciplinary expertise.

Overwhelming evidence now suggests that the brain is characterized, at least in part, through these perspectives. As we embrace analyses designed for complexity, we should be careful to appreciate the fine line between simplification for conceptual clarity versus oversimplification that misrepresents the true phenomenon under study. For example, researchers tend to focus on only part of a network for visualization and interpretation when performing network analyses. While understanding the relative contributions of features is helpful, we may not be at the stage where we can fruitfully pick and choose and should strongly consider casting a wide net to retain as much potentially relevant information as possible, then carefully whittling down our models from there. Happily, advances in data and computation now afford us the opportunity to capture higher dimensional information and build larger models than ever before, albeit with new challenges in validating and making sense of this information.

Furthermore, given the number of open questions, it may be prudent to blend theory- and data-driven approaches. There is much excitement about, for example, machine learning in human neuroscience. However, less collective attention has been paid to a theoretical characterization of the human brain from a complex systems perspective (besides a fairly utilitarian approach towards building brain networks), suggesting that more theory-driven investigations are needed. At the same time, we should grow comfortable with the notion that we may never fully understand some of the models we build.

## 6 Concluding Remarks

We want to acknowledge the substantive contributions of localizationism to the field, having used these approaches in our own work. At the same time, it is necessary to reflect on the limitations of these methods and provide constructive criticism to advance the field and future discovery.

It is not a new observation that localizationism and reductionism have limitations. Indeed, as discussed above, concerns were raised at least decades ago. Yet it remains common to search for specific regions associated with a complex behavior or disorder. Thus, recent empirical challenges to localizationism are not only as relevant as ever, but also further serve to tangibly illustrate the scope of these limitations and motivate better practices.

We are not alone in facing this challenge. Other fields that deal with the intricacy of unraveling the biological and psychological bases of behavior are in the same boat. Animal researchers have recently highlighted the limitations of one-to-one brain-behavior mapping techniques (Tremblay et al., 2022) and advocated for the potential of new multi-region recording techniques to uncover collective phenomena (Machado et al., 2022). As in neuroimaging, mass univariate analyses are widespread in genetics, but researchers have pushed for methods that better account for the distributed nature of the signal (van der Meer et al., 2020). Mental health and psychology research has undergone a revolution in transcending single disorder categories in favor of complex, transdiagnostic networks of symptoms (Hofmann et al., 2016). The shared shift in perspective promoted here—one that embraces complexity—is expected to bring about additional challenges of a magnitude we have not yet encountered. Yet the potential for more reproducible and accurate discovery makes such a shift necessary for us all to move forward.

## Footnotes

1 Although the examples discussed here are primarily circumscribed to neuroimaging, we believe that the findings from this body of work have strong implications for other modalities of human neuroscience research more broadly and thus often refer to “human neuroscience” accordingly throughout this manuscript.

2 Note that this is a broader topic than we will address in the present manuscript, and a subject of active debate among philosophers of science. However, it is important to indicate where neuroscience research falls in this debate given that it underlies other influential conceptual assumptions such as localizationism.

3 Namely, it is common to perform exploratory mass univariate tests across the brain and then threshold resulting statistical maps based on a multiple testing-corrected significance level.

4 Note that multiple realizability also provides a philosophical foundation for*emergentism*(i.e., the philosophical view that complex phenomena emerge from the interactions of simpler components in a system).

5 In practice, there are many types of “collective properties,” and it is helpful to state which is under investigation (e.g.,Pessoa, 2023, encourages us to start by contemplating “system interactivity”).
